# Great Spotted Cuckoo Fledglings Often Receive Feedings from Other Magpie Adults than Their Foster Parents: Which Magpies Accept to Feed Foreign Cuckoo Fledglings?

**DOI:** 10.1371/journal.pone.0107412

**Published:** 2014-10-01

**Authors:** Manuel Soler, Tomás Pérez-Contreras, Juan Diego Ibáñez-Álamo, Gianluca Roncalli, Elena Macías-Sánchez, Liesbeth de Neve

**Affiliations:** 1 Departamento de Zoología, Facultad de Ciencias, Universidad de Granada, Granada, Spain; 2 Grupo Coevolución, Unidad Asociada al Consejo Superior de Investigaciones Científicas (CSIC), Universidad de Granada, Granada, Spain; 3 Department of Biology, Terrestrial Ecology Unit, Ghent University, Gent, Belgium; University of Lausanne, Switzerland

## Abstract

Natural selection penalizes individuals that provide costly parental care to non-relatives. However, feedings to brood-parasitic fledglings by individuals other than their foster parents, although anecdotic, have been commonly observed, also in the great spotted cuckoo (*Clamator glandarius*) – magpie (*Pica pica*) system, but this behaviour has never been studied in depth. In a first experiment, we here show that great spotted cuckoo fledglings that were translocated to a distant territory managed to survive. This implies that obtaining food from foreign magpies is a frequent and efficient strategy used by great spotted cuckoo fledglings. A second experiment, in which we presented a stuffed-cuckoo fledgling in magpie territories, showed that adult magpies caring for magpie fledglings responded aggressively in most of the trials and never tried to feed the stuffed cuckoo, whereas magpies that were caring for cuckoo fledglings reacted rarely with aggressive behavior and were sometimes disposed to feed the stuffed cuckoo. In a third experiment we observed feedings to post-fledgling cuckoos by marked adult magpies belonging to four different possibilities with respect to breeding status (i.e. composition of the brood: only cuckoos, only magpies, mixed, or failed breeding attempt). All non-parental feeding events to cuckoos were provided by magpies that were caring only for cuckoo fledglings. These results strongly support the conclusion that cuckoo fledglings that abandon their foster parents get fed by other adult magpies that are currently caring for other cuckoo fledglings. These findings are crucial to understand the co-evolutionary arms race between brood parasites and their hosts because they show that the presence of the host's own nestlings for comparison is likely a key clue to favour the evolution of fledgling discrimination and provide new insights on several relevant points such as learning mechanisms and multiparasitism.

## Introduction

Parental behaviours that enhance the fitness of offspring while provoking a cost to the parents are considered to be parental care [1]. Parental care is costly, not only in terms of time and energy, but also because parental investment in current reproduction implies a reduction in the number of future offspring [1], [2]. Natural selection also penalizes individuals that provide costly parental care to non-relatives. Therefore, parental investment theory predicts that parents should have adaptations allowing them to both optimize the balance between investment in current and future reproduction and to recognize kin. For instance, in many taxa it is well known that parents usually favour offspring of higher reproductive value [3]–[5] and that parents become more insensitive to begging signals by their offspring when they are ready to initiate a second breeding attempt [6]. Furthermore, offspring desertion occurs when fitness costs related to investment in the current brood exceed the expected fitness benefits [7]–[9], for example in response to partial egg predation [7], [9], [10]–[12]. In addition, males reduce parental investment in cases of reduced certainty of paternity [13]–[16].

Many parental care behaviours are susceptible to parasitism resulting in a huge variety of interactions in which various combinations of nest, food and offspring care are parasitized [17]. In fact, parental care parasitism by unrelated individuals is widely distributed within the animal kingdom (reviewed in [17]).

Alloparental care could be considered a type of parental-care parasitism in which adult animals feed unrelated juveniles [17]. This behaviour, as it occurs with nest switching [18], has been treated by the literature as adoption behaviour by foster parents. However, since alloparental care behaviours are promoted by the young themselves rather than by the foster parents, they should be considered cases of parental-care parasitism [17].

Alloparental care is especially frequent at the intraspecific level in cooperatively breeding species, in which the parasitized individual is related to the juveniles and receives benefits from inclusive fitness [17], [19]. However, it is also very frequent in birds at the interspecific level in situations of brood parasitism. When brood parasitic fledglings leave the host nest, they continue being fed by their foster parents [20]–[23]. This is not surprising because host foster parents could learn the begging calls of a parasitic nestling at the end of the nestling period, and later, continue feeding it because they had learnt the vocal signature of that fledgling as one of their own nestlings [24]–[28].

However, reports of brood-parasitic fledglings being fed by individuals other than their foster parents, or even individuals from a different host species are also common [20], [22], [29]–[38], especially for the pallid cuckoo (*Cuculus pallidus*; [38]) and the great spotted cuckoo (*Clamator glandarius*; [36], [37]). However, all these reports were of anecdotic nature and based on very few observations [38]. Still, observations of parasitic fledglings being fed by individuals other than their foster parents could be explained by the hypothesis that the parasitized parents were tricked into feeding because they were exposed to the proper stimulus (i.e. a begging fledgling), which is the most important prerequisite for expressing feeding behaviour [39].

The great spotted cuckoo is a non-evictor brood parasite that uses the magpie (*Pica pica*) as its primary host. The nestling period of great spotted cuckoo chicks is considerably shorter than that of magpie chicks (between17–22 days and 23–24 days, respectively for cuckoos and magpies [40]). Great spotted cuckoo fledglings have a post-fledging dependence period that is highly variable (between 25 and 59 days, [21]). Between one and three weeks after leaving the nest, usually join in groups that are communally fed by more magpies than those involved in rearing those cuckoos in the nest [37], [41]. In a recent experimental study, we found that great spotted cuckoo fledglings that had been reared together with magpie nestlings in the same nest were disadvantaged (in terms of feeding patterns) by magpie adults compared to cuckoo fledglings that had been reared in only cuckoo broods [23]. They were fed less frequently than those reared in only cuckoo broods, and magpie adults approached less frequently to feed cuckoos from mixed broods than cuckoos from only cuckoo broods [23]. These, probably undernourished, great spotted cuckoo fledglings might abandon their less-efficient foster parents and join other cuckoo fledglings, to obtain a higher feeding rate in a communal fed group [37]. This ability to look for more efficient caregivers has frequently been reported in some cooperative species [42]–[44] but, as far as we know, it has never been found in fledglings of any other brood parasite species. The study of the relationships between brood parasites and their hosts during the post-fledgling period is very important because of two reasons. First, because hardly anything is known about the post-fledging period in brood parasites and hosts [45] in spite that this period of care is critical for juvenile survival [46]. Second, coevolutionary adaptations and counter-adaptations can evolve at any stage of the breeding cycle, including the fledgling stage [22], [45], [47], [48] (host defences have also been found at the nestling stage in several brood parasite – host systems [49]–[51]; reviewed in [52], [53]). The clearest example of an arms race at the fledgling stage comes from the bay-winged cowbird *Agelaioides badius* that feeds fledglings of its specialist parasitic screaming cowbird *Molothrus rufoaxillaris*, which visually and vocally mimic host fledglings, but it refuses to feed non-mimetic fledglings from a generalist brood parasite, the shiny cowbird *M. bonariensis* [22], [47].

The main aim of this study is to study in depth the relationships between great spotted cuckoos and their magpie hosts during the post-fledgling stage. We were especially interested in the most surprising behaviour reported during this stage; i.e. that magpie adults sometimes feed cuckoo fledglings that have abandoned their foster parents. We performed three different experiments during four breeding seasons to answer the following crucial questions related to this behaviour: (1) is obtaining food from magpies other than their foster parents a frequently used strategy by cuckoo fledglings or are they only anecdotic cases as reported in brood parasitic fledglings of other species? (2) Which magpies accept to feed foreign cuckoo fledglings that beg for food?

## Material and Methods

### Ethics Statement

Research has been conducted according to relevant Spanish national (Real Decreto 1201/2005, de 10 de Octubre) and regional guidelines. All necessary permits were obtained from the Consejería de Medio Ambiente de la Junta de Andalucía, Spain. Approval for this study was not required according to Spanish law because it is not a laboratory study in which experimental animals have to be surgically manipulated and/or euthanatized. Our study area is not protected but privately owned, and the owners allow us to work in their properties. This study did not involve endangered or protected species. The great spotted cuckoo is included in both Spanish national (R. D. 139/2011, 4 February) and regional (D. 23/2012, 14 February) lists of species under special protection, but not in the catalog of endangered species of any of the two entities.

Plastic patagial wing tags used to mark adult magpies cause no damage to the individuals (e.g. [54]). After releasing the magpies, we made observations in each territory to ensure that the captured individuals flew correctly, continued in the territory and maintained their nests. None of the adult magpies showed problems to fly or abandoned the nest or their nestlings because of the capture and marking process. Cross-fostering manipulations were made by carefully transporting the nestlings in an artificial cotton nest lined with tissues, maintaining the temperature in the car between 25–30°C. Cross-fostering per se does not affect nestlings or host parents' behaviour [40], [41], [55]. In some cases in which we took one great spotted cuckoo chick that was alone in the nest we left another cuckoo chick of similar age from a multiparasitized nest to avoid nest abandonment. Transmitters were attached using the leg-loop harness method, which has been demonstrated to be effective without causing skin or plumage damage, or interfering with behaviour [56], [57]. Attaching the transmitters several days before the fledglings leave the nest allows the nestlings to become accustomed to it and allow the harness to fit the nestling body.

### Study species, study area and general field methods

The relationships between great spotted cuckoo fledglings and adult magpies have been extensively studied [21], [23], [27] and it has been observed that the feeding of one fledgling great spotted cuckoo by more than two magpies is frequent [37].

This study was carried out during the breeding seasons of 2007, 2010, 2011 and 2012 in a population of great spotted cuckoos located in the Hoya de Guadix in southern Spain (37° 10′ N, 3° 11′ W; 1000 m.a.s.l.). This area is a high-altitude plateau (approx. 1000 m a.s.l.) with extensive non-cultivated areas, cereal crops (especially barley), some areas with dispersed holm-oak trees (*Quercus rotundifolia*) and groves of almond trees (*Prunus dulcis*) and pines (*Pinus halepensis* and *P*. *pinaster*), in which magpies build their nests [58]. The land in the Hoya the Guadix is privately owned by many different landowners, but most fields are not fenced and so the magpie territories are freely accessible. Most proprietors allow us to follow up nests on their land during the breeding season.

Occurrence of brood parasitism was frequent in the study area with 56.8% of magpie nests parasitized by great spotted cuckoos for the period 2008–2012 [23].

Every year we searched for new magpie nests during the complete breeding season (mid March – early June) and recorded the main breeding data, i.e. laying date, clutch size, presence and number of great spotted cuckoo eggs and the number of great spotted cuckoos and magpies that successfully left each nest.

### Experiment 1: translocation of fledglings

During June-July of 2007, we captured great spotted cuckoo fledglings of about 40 days old (i.e. about 20 days after fledging; with wings and tails completely developed) using mist nets and provided them with a radio-transmitter (Holohil PD-2, weight: approximately 4.5 g, back-pack harness included, range of 1000 m and a battery life of 14–26 weeks). Transmitters were attached using the leg-loop harness method [59]. Further details about radio-transmitters and attaching method can be found in [23]. These fledglings were randomly assigned to one of the two following treatments: (i) experimental treatment: fledglings were retained temporally within a cloth bag while we transported them by car to a different area with cuckoo fledglings (a mean ± SD of 7.00±3.51 km away from their original area) where they were released), and (ii), control treatment: captured fledglings were also retained temporally within a cloth bag, and after about 15 minutes they were released in the same area where they were captured. All captures and releases were done with good weather conditions and in the presence of no potential predators of cuckoo fledglings (i.e. raptors).

Every 3–5 days we went to search for the released fledglings with a reception antenna (Televilt (now Followit) O-5/8, receptor RX-98H). Once close enough to the signal (about 150 meters) we searched visually for the activity of the individual using binoculars. If we failed to detect the fledgling after 15 min, despite the close signal, we approached the place in order to look for the cuckoo fledgling more closely and determine its situation (if it was dead or alive). We followed the activity of all marked cuckoo fledglings for two weeks, a period of time long enough to allow us to conclude that the fledgling survived in the new situation (i.e. after the experimental translocation).

During our inspections of each fledgling we did not wait to make observations of feedings by magpies, however, we assume that survival of the translocated fledglings implied that magpies were feeding them. This assumption is based on four points. First, great spotted cuckoo fledglings have never been seen feeding themselves [21, 23, this study]. Second, magpies other than foster parents have frequently been reported feeding great spotted cuckoo fledglings [36], [37]. Third, cuckoo fledglings could not suddenly change to feed themselves given that fledglings of altricial species need a long period to achieve foraging skills (e.g. [46]). And fourth, the assumption has been supported by Experiment 3 (see below).

The translocation experiment was done to test the hypothesis that cuckoo fledglings frequently use the strategy to obtain food from magpies other than their foster parents, instead of being only anecdotic cases of alloparental feedings. Thus, we predicted that well developed fledglings translocated to a different area from their rearing territory, should be able to survive equally well than those that remain in their natal area (Prediction 1).

### Experiment 2: playback-stuffed-cuckoo presentation

We actively searched for adult magpies and great spotted cuckoo fledglings in the study area at the end of the breeding seasons of 2011 and 2012. Once adult magpies or cuckoo fledglings were detected in the field, we observed the location for a variable period of time from a distance of about 200 m using binoculars to determine the number, species (cuckoo or magpie) and age (adult or fledgling) of individuals. When this information was collected we presented a stuffed great spotted cuckoo fledgling (6 different stuffed-cuckoo fledglings mounted with the beak closed and in a non-begging display), with begging calls (4 different playbacks; 60 s of begging and 45 s of silence) for 30 min in the following two situations: in the presence of adult magpies that were together with cuckoo fledglings alone (experimental treatment) or in the presence of adult magpies accompanied by only magpie fledglings (i.e. family group; control treatment). We avoided testing the same individuals by doing the experiments in clearly separated locations (more than two km).

The experimental procedure consisted in the placement of a stuffed-cuckoo fledgling in a visible location (usually on the ground in an open area) close to the group of birds but far enough to avoid frightening them (between 100–200 m). We placed the playback device on the ground, as close as possible to the stuffed cuckoo, covered with a camouflage fabric. The playback device consisted in a MP3 device, an amplifier and two speakers powered by a battery. We observed the behaviour of adult magpies for 30 min from a hidden location about 200 m away from the experimental setup using binoculars and recorded the following variables: latency (the time elapsed between the start of observation and the first adult magpie approaching the stuffed cuckoo closer than 50 m), the number of different magpies approaching the stuffed cuckoo, the number of times that magpies approached, and the approach behaviour (negative, positive or neutral). An approach was classified as negative when the adult magpie showed an aggressive behaviour against the stuffed cuckoo usually involving scolding calls and/or flying over it repeatedly. Positive approaches involved carrying food to the stuffed cuckoo, while neutral approaches were attributed to those observations where the adult magpies ignored the playback and the stuffed cuckoo.

This playback-stuffed-cuckoo experiment was performed to test the hypothesis that cuckoo fledglings that abandon their foster parents are only fed by other magpies that are already caring for cuckoo fledglings. This hypothesis is based on previous findings showing that cuckoo fledglings reared together with magpie fledglings were fed less frequently than those reared in only cuckoo broods [23]. We predicted that the stuffed cuckoo will receive neutral or aggressive responses by magpies attending magpie fledglings, and more positive responses by magpies attending cuckoo fledglings (Prediction 2).

### Experiment 3: non-parental-feeding observations

During the breeding season of 2012 we found a total of 133 magpie nests. We assigned each nest to one of the following experimental treatments depending on the composition of the brood: (i) only cuckoos (1–3 great spotted cuckoo nestlings), (ii) only magpies (2–5 magpie nestlings), and (iii) mixed broods (one cuckoo and one or two magpie nestlings). These experimental groups were created by cross-fostering 1 or 2 day old nestlings by carefully transporting them to the corresponding nest. For further information on the cross-fostering manipulation see [40], [55], [60]. We decided to carry out this cross-fostering manipulation because in naturally parasitized nests, the parasitic chicks usually outcompete their host nestmates with a series of adaptations [58], [61], [62]. Thus, this manipulation was necessary in order to ensure the survival of both parasite and host nestlings in the same nest until fledging.

We tried to capture as many breeding adult magpies as possible at experimental nests during the entire breeding period (from April to June). In order to do so, we used decoy traps (with a live magpie inside; [63]), which were located in a visible place near the nest. Once captured, adult magpies were marked with numbered metal rings (Ministerio de Agricultura - ICONA) and two patagial wing tags (4 cm length x 3 cm width) with a unique alphanumeric combination. The wing tags were made of PVC fabric of high resistance that causes no damage to the individuals (e.g. [54]) and allows their visual identification from a distance. We managed to capture a total of 66 adult magpies (34 females, 32 males) belonging to a total of 39 different territories: 27 complete pairs (male and female) and 12 cases in which we captured only one of the two adults (Table 1). We measured standard biometrical parameters (weight, wing, tail and tarsus lengths) to identify the sex of each individual [64]. We likewise checked for the presence of a brood patch in incubating females. Additionally, we used behavioral differences between sexes during the breeding season to confirm the sex of pair members [65], [66].

**Table 1:** Sample sizes of the total number of marked and observed individuals (adult magpies and cuckoo nestlings) in relation to their breeding status.

Territory type	Marked		Observed	
	Adults	Fledglings	Adults	Fledglings
Only cuckoo	32 (18)	41 (27)	19 (11)	19 (15)
Only magpie	5 (3)	NA	0 (0)	NA
Mixed broods	11 (6)	6 (6)	6 (4)	4 (4)
Failed broods	18 (12)	NA	0(0)	NA

The number of nests to which the individuals belong are indicated between brackets. NA  =  Not applicable.

We regularly (every 2–3 days) checked all nests and some days before the expected date when cuckoo nestlings leave the nest, we equipped them with a radio-transmitter (Holohil PD-2, see Experiment 1) and patagial wing tags, similar to those attached to adult magpies, but smaller (3 cm length x 2 cm width). Transmitters were attached using the same method described in Experiment 1. A total of 47 great spotted cuckoo nestlings from 33 different nests were equipped with both radio-transmitters and patagial wing tags (Table 1). Six of them were from mixed broods and 41 from “only cuckoo” broods (Table 1).

All nests were monitored in detail until all chicks had left the nest, so we could determine the breeding experience of each magpie pair during the current breeding season (hereafter referred to as “breeding status”, Table 1): (i) only cuckoo: adults that have raised exclusively cuckoo chicks until fledging; (ii) mixed broods: adults that have raised at least one cuckoo and one magpie chick until fledging; (iii) only magpies: adults that only raised magpie chicks until fledging; (iv) magpies that failed to raise any chicks until fledging (due to natural causes; e.g. predation).

We carried out an intensive observation schedule of post-fledging feeding events to radio-tracked cuckoos (about 350 hours) following the methodology described in [23]. Basically it consisted in locating radio-tracked cuckoo fledglings using the radio-tracking method (3 element hand-held antennas O-5/8, receptor RX-98H (Televilt, now Followit)) and observing them carefully from the distance.

When a fledgling was detected, we noted its identity (i.e. the frequency of its radio-transmitter) and double-checked it through the observation of the alphanumeric combination of its wing tags. We continuously observed the focal fledgling until we lost sight of the individual. We obtained reliable observations from 23 fledglings, belonging to 15 only cuckoo territories and 4 mixed territories (Table 1), i.e. the fledgling was observed for more than two hours on each observation day (mean ± SE: 136.1±12.6 minutes: *N* = 152 observations). In each feeding event we carefully recorded the identity of the feeding adult magpie (i.e. the number of its wing tag). A total of 374 feeding events were observed in which 25 marked adult magpies were involved (Table 1). We made a strong effort to mark as many adult magpies as possible but not all individuals involved in the observed feeding events were marked birds. We considered a feeding interaction as “non-parental” only when both foster parents of the focal cuckoo fledgling were marked and the fledgling was fed by another adult magpie (either marked or not) than its foster parents, or when its foster parents fed another (marked or not) cuckoo fledgling that was not the one raised by them. In case that not both foster parents were marked, we only considered a feeding as non-parental when the focal fledgling was fed by another marked adult magpie that was not its foster parent. All the observations were made during the most active periods, i.e. from sunrise to 11 a.m. and from 6 p.m. until sunset.

With this experiment, we want to test two predictions. First, based on the hypothesis presented in experiment 2, we predict that non-parental feedings will be mainly provided by magpies caring for only cuckoo fledglings (Prediction 3a). Another non-mutually exclusive hypothesis can also be considered: magpies that failed to fledge any chick could also contribute to provide non-parental feedings to fledgling cuckoos. This hypothesis is based on two ideas: (1) only the proper stimulus (i.e. a fledgling cuckoo begging for food) is enough to provoke alloparental feedings [39], (2) caring for even unrelated fledglings could increase the probabilities of maintaining or acquiring breeding status [67]. This hypothesis predicts that we should find unsuccessful magpies (i.e. those that reared no nestlings until fledging) providing non-parental feedings to fledging cuckoos (Prediction 3b).

### Statistical analyses

To analyze the data of our translocation study (Experiment 1), we used a generalized linear model (GLZ) to test if the probability of survival after two weeks (binomial error) was different between cuckoo fledglings from the two translocation groups (fixed factor).

To analyze data collected from the playback-stuffed-cuckoo experiment (Experiment 2), we carried out a generalized (GLZ) or general linear model (GLM), depending on the nature of the response variables. We considered the “approach behaviour” (multinomial distribution) and the number of different magpies approaching to the stuffed cuckoo (Poisson distribution) as the response variables for the generalized linear models. Time of latency and the total number of approaches fitted a normal distribution after transformation (log or sqrt) and were the response variables for the general linear models. Treatment (i.e. adult magpies in family groups either with magpie or cuckoo fledglings) and year were included as nominal independent variables, while the number of adult magpies present at the beginning of the experiment was considered as a covariable for all analyses.

The observations on non-parental feedings were analysed from both the adult's and the fledgling's point of view (see Results). We pooled information from different individuals from the same territory, either adults or cuckoo chicks, to avoid pseudoreplication (i.e. each nest is considered as an independent unit). We calculated the ratio of non-parental feedings out of all feeding observations, and analyzed with a General Linear Model (GLM) if the proportion of non-parental feedings differed between nests that only raised cuckoo chicks and those that raised mixed broods (i.e. the two types of territories in which both adults and fledglings have been observed; Table 1). In addition, we used a repeated measures ANOVA to investigate if the total number of non-parental and parental feeding events per hour differed between male and female adult magpies (sex and type of feeding as within factors). This final analysis was done using only adults of only cuckoo territories as they were the only adult magpies involved in non-parental feeding events (see Results).

All analyses were made with STATISTICA 7.0 (StatSoft Inc. 2001–2004).

## Results

### Experiment 1: translocation of fledglings

We managed to capture and radio-track 15 different great spotted cuckoo fledglings (7 control, 8 translocated; see Database S1). We only found one marked fledgling dead in each treatment group. Cuckoo fledglings survived equally well when released in their own area or when moved to another area (GLZ, χ^2^_1_ = 0.01, *p* = 0.91). This result is in agreement with Prediction 1 (i.e. translocated fledglings should be able to survive equally well than those that remain in their natal area).

### Experiment 2: playback-stuffed-cuckoo presentation

We managed to present the playback and stuffed cuckoo in 56 different magpie territories (11 territories with only magpie fledglings, 45 territories with only cuckoo fledglings) which is the sample size for all analyses except for time of latency (N = 42; see Database S2).

**Figure 1:**
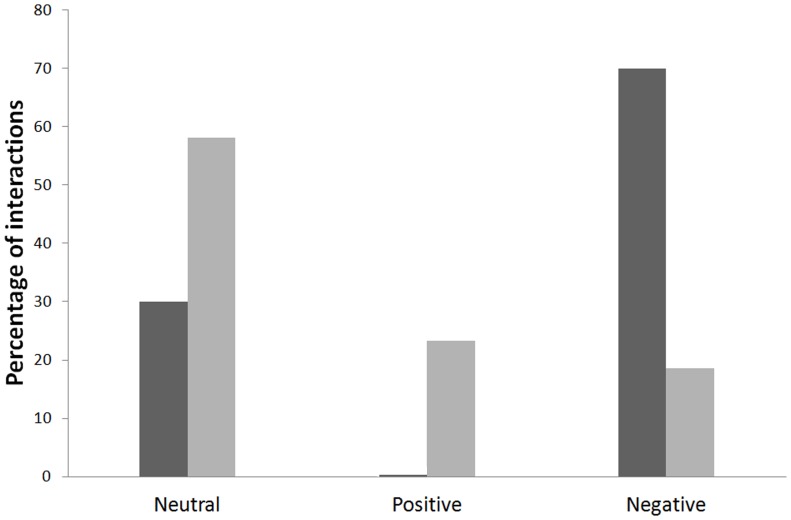
Adult magpies' “approach behaviour” (neutral, positive or negative) to the presentation of the stuffed cuckoo depending on the presence of other cuckoo fledglings (soft grey) or only magpie fledglings (dark grey). See Material and Methods section for a detailed explanation for each type of “approach behaviour”.

We found a significant difference in “approach behaviour” by adult magpies against the stuffed cuckoo between treatments (GLZ χ^2^_2_ = 12.60, *p* = 0.002; *N* = 56). The frequency of negative behaviours against the stuffed cuckoo was much higher when adult magpies were in family groups with only magpie fledglings (aggressive response in 70% of the trials) than when they were observed together with cuckoo fledglings (only in 19% of the trials; Fig. 1). In addition, feeding trips to the stuffed-cuckoo model were only observed when adult magpies were together with great spotted cuckoo fledglings (23% of the trials; Fig. 1). These results strongly support Prediction 2 (i.e. the stuffed cuckoo will receive a neutral or aggressive response by magpies attending magpie fledglings, whereas magpies that were attending cuckoo fledglings will provide a more positive response).

We did not find any significant differences between treatments in the time of latency (GLM *F*_1, 37_ = 0.04, *p* = 0.84), the total number of approaches (GLM *F*_1, 51_ = 0.26, *p* = 0.61) or the number of different adult magpies approaching the stuffed cuckoo (GLZ χ^2^_1_ = 0.54, *p* = 0.46). The number of adult magpies present in the area at the beginning of the experiment was positively related to the total number of approaches and the number of different magpies approaching (GLM, *F*_1, 51_ = 5.60, *p* = 0.02; and GLZ, χ^2^_1_ = 23.26, *p*<0.0001, respectively).

### Experiment 3: non-parental-feeding observations

**Figure 2:**
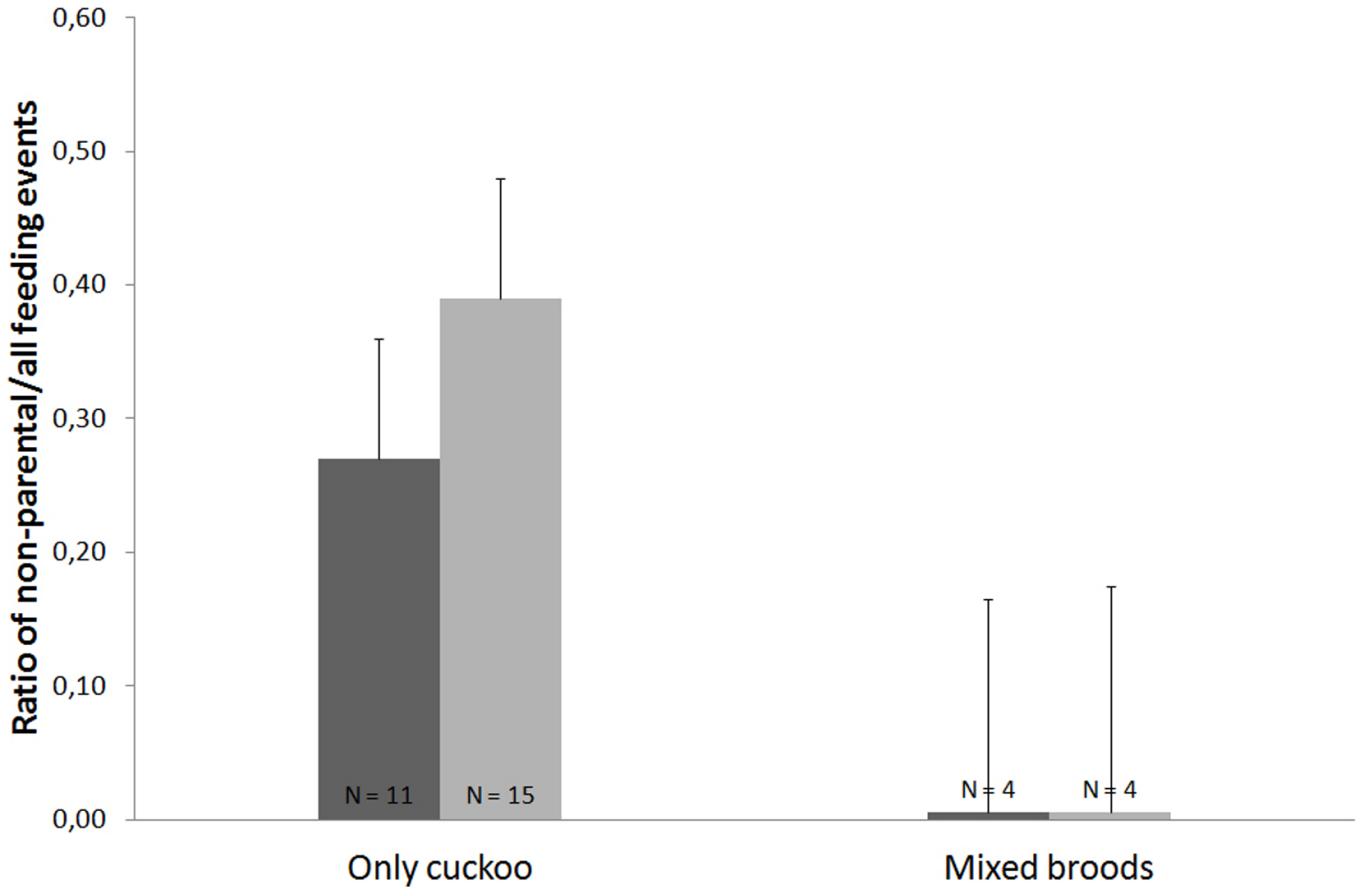
Ratio of non-parental feeding events out of all observed feedings provided by adult magpies (dark grey) or received by cuckoo fledglings (soft grey) for each territory type. Note that “only magpie” and “failed broods” territories are not represented as adults of these kinds of territories were never observed feeding great spotted cuckoo fledglings. Data represented are Least Square Means ± SE.

Data obtained in this experiment can be found in Database S3. From the adults' point of view (i.e. feedings provided to cuckoo fledglings by marked adult magpies), there were no significant differences in the ratio of non-parental feedings provided between adults that raised only cuckoo broods and those that raised mixed broods (GLM *F*_1, 13_ = 2.16, *p* = 0.17), although all (100%) non-parental feedings corresponded to adults that raised only cuckoo broods (Fig. 2) (which supports Prediction 3a). In fact, if we consider only those territories in which we observed non-parental feedings (N = 5), we found that these events involved between 22% and 100% of all the observed feedings (N = 201 feedings, mean 58.6±12.5% non-parental feedings). In contrast, we never observed adult magpies from families that raised only magpie fledglings (in agreement to Prediction 3a) or from families with failed breeding events providing non-parental feedings to any of the observed cuckoo fledglings (contrary to Prediction 3b).

From the fledglings' perspective (i.e. feeding interactions observed to marked cuckoo fledglings), we found that non-parental feeding events involved a marginally significantly higher proportion of the feedings to fledgling cuckoos from only cuckoo broods compared to those that were raised together with magpie fledglings (GLM *F*_1, 17_ = 4.30, *p* = 0.05; Fig. 2). Again, all (100%) observed non-parental events involved fledglings from only cuckoo territories. Furthermore, if we consider the territories in which we observed cuckoo fledglings receiving non-parental feedings (N = 9), we found that a mean of 59.1±10.3% (range 10.7% to 100%) of the feedings (N = 184) consisted of non-parental feedings.

**Figure 3:**
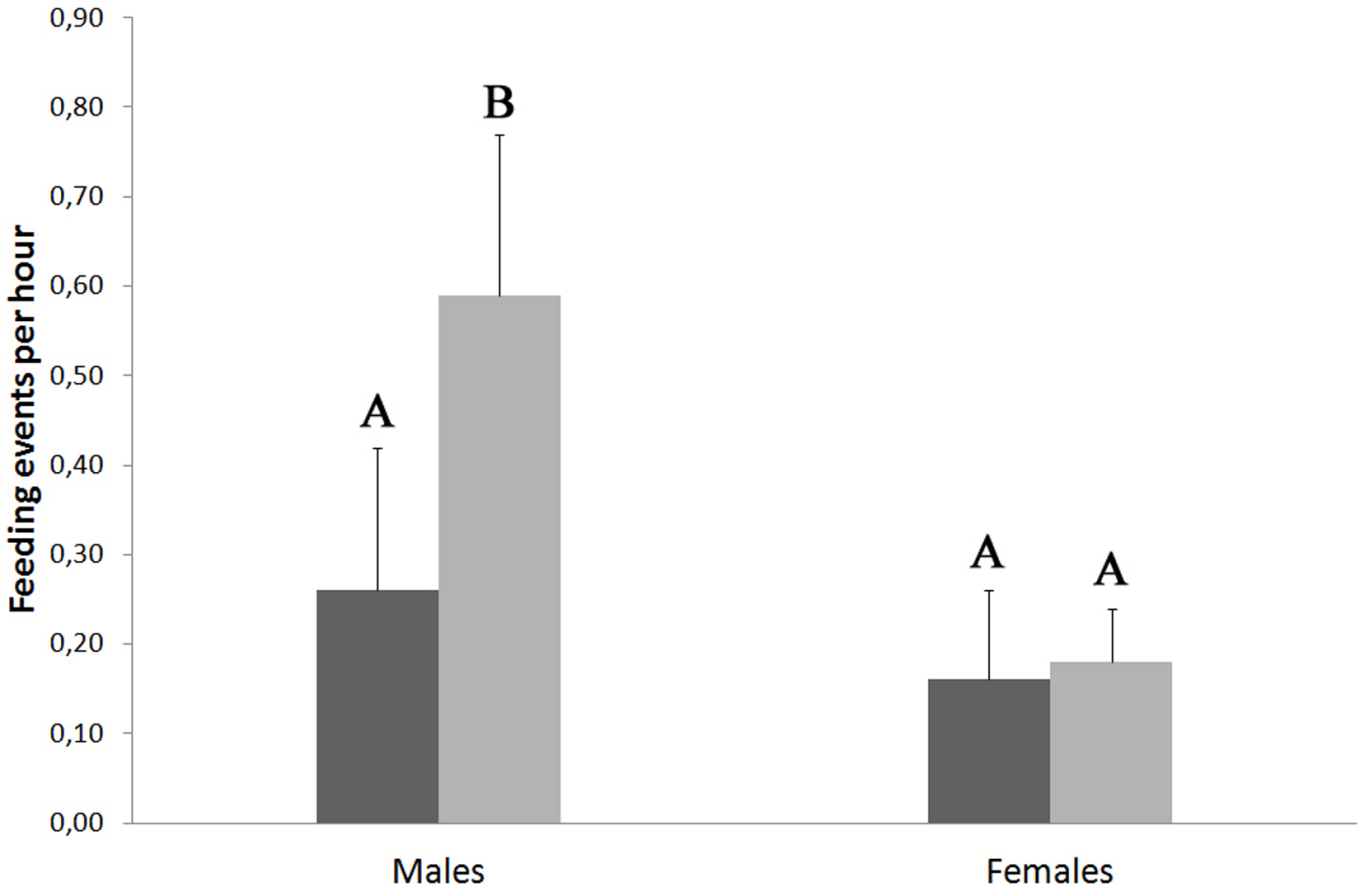
Sex differences for adult magpies in the number of non-parental (dark grey) and parental (soft grey) feedings per hour provided to great spotted cuckoo fledglings.

Regarding sexual differences, we did not find significant differences in the total number of feedings provided by adult magpies to cuckoo fledglings between males (0.42±0.19 events/h) and females (0.17±0.07 events/h; RM-ANOVA F_1, 10_ = 3.10, *p* = 0.11). However we found a marginally non-significant effect for the interaction type of feeding and sex (RM-ANOVA *F*_1, 10_ = 3.37, *p* = 0.09) indicating that male, but not female magpies, tend to feed their own cuckoo fledglings more frequently than other unknown cuckoos (Tukey HSD posthoc: *p* = 0.07; Fig. 3). Furthermore, males significantly feed more actively their cuckoo fledglings than females (Tukey HSD posthoc: *p* = 0.03; Fig. 3).

## Discussion

The translocation experiment (Experiment 1) has demonstrated that cuckoo fledglings survived well when moved far away from their natal territories, which indirectly suggest that they managed to be fed by other magpies than their foster parents (those that reared them in the nest). In fact, our third experiment showed that non-parental feeding interactions (i.e. feedings provided by magpies other than their foster parents) involve a high percentage (43%) of all observed feedings to cuckoo fledglings. These data support the hypothesis that obtaining food from magpies other than their foster parents can be a successful and frequently used strategy by some great spotted cuckoo fledglings.

It is well known, and considered to be adaptive, that adult birds frequently attack unrelated offspring that beg for food [68], [69]. Then, why do brood parasitic fledglings manage to get fed by other adults different than their foster parents? The results of our translocation experiment imply that cuckoo fledglings have adaptations that enable them deceive magpies into feeding them. The existence of such adaptations has also been suggested by Eastzer et al. [70] who found that barn swallows (*Hirundo rustica*) that had raised nestlings from three different species only continued to feed the brood parasitic brown-headed cowbird (*Molothrus ater*) after leaving the nest.

A key factor of this potential adaptation is begging behaviour, which is more exuberant in fledglings of brood parasites than in fledglings of host species [20], [37], [70], [71] and could have an important role in attracting host attention. Another aspect related to begging is vocal mimicry, because host ability to discriminate parasitic fledglings would select for mimetic begging calls in brood parasite fledglings [22], [47]. However, this does not seem to be the case in great spotted cuckoo fledglings because their begging calls are very different from those of magpie fledglings (own observations), and neither during the nestling stage vocal mimicry exists in this brood parasite species [72].

The fact that some magpies after hearing the begging calls of great spotted cuckoo fledglings approached the stuffed-cuckoo fledgling disposed to feed it indicates that the begging calls are the stimuli responsible for the fact that magpies other than their foster parents feed them. Although it could be possible that magpies cue on the begging calls to locate the fledgling and then decide whether to feed it or not based on visual cues too. Two pieces of evidence support the former statement. First, in a first try-out of performing Experiment 3, the same stuffed-cuckoo fledglings (which were mounted with the beak closed and in a non-begging display; see Methods) did not provoke any approaching by magpies, neither in absence of the begging calls (*N* = 8) nor when accompanied by bad quality recordings of fledgling begging calls (i.e. recordings taken at long distance without the appropriate recording material, *N* = 18) (Soler et al. unpublished). Second, begging call is the most important component of communication between brood parasite fledglings and their foster parents [20], [22], [37].

However, interestingly, the response of magpies to the playback-stuffed cuckoo (Experiment 2) depended on the social situation: adult magpies showed some level of predisposition to feed the stuffed-cuckoo fledgling only when they were observed together with cuckoo fledglings (i.e. magpies that were attending a group of cuckoo fledglings). Adult magpies that were in family groups together with magpie fledglings never approached to feed the stuffed cuckoo and the response against it was frequently aggressive (Fig. 1). These results are in agreement with previous findings. Magpies rearing a cuckoo nestling accepted and fed other cuckoo nestlings experimentally introduced into their nests during the last phase of the nestling period, while magpies from non-parasitized nests frequently were reluctant to feed the experimental cuckoo nestling, and even, sometimes rejected it [27]. In addition, after leaving the nest, cuckoo fledglings that had been reared together with magpie fledglings were less intensely defended than their magpie nestmates, less frequently fed than cuckoo fledglings reared in “only cuckoo broods”, and more importantly in relation to this study, magpie adults approached to feed cuckoos from mixed broods less frequently than cuckoos from “only cuckoo broods” [23]. These results suggest that the presence of host's own nestlings for comparison may be a crucial clue favouring the evolution of fledgling discrimination [22, 23, 47, this study]. An effect of opportunities for hosts to compare own and foreign chicks on nestling discrimination was also suggested [73], [74], however, recent reviews have showed that such an effect is not important [52], [75].

Our playback-stuffed-cuckoo presentation study (Experiment 2) has shown that only some magpies that are already caring for cuckoo fledglings are willing to feed the stuffed cuckoo (i.e. unknown cuckoo fledglings that try to join their group of cuckoos). Which magpies are those that have the motivation to feed foreign cuckoo fledglings that beg for food? This is a very important question for the understanding of the evolution of the arms race between great spotted cuckoos and their magpie hosts at the fledgling stage. Our study on feeding interactions (Experiment 3) was conducted in a population of previously marked magpies to answer this question and we found, although with small sample sizes, that 100% of non-parental feeding events were provided by magpies that were caring only for cuckoo fledglings (Fig. 2), which support Prediction 3a. This result confirms previous findings obtained in Experiment 2. Thus, we can conclude that possibly undernourished great spotted cuckoo fledglings that abandon their foster parents and join a group of other fledglings [23] are fed by adult magpies that only have reared cuckoos. The alternative hypothesis that unsuccessful magpies that fail to rear any chick could also provide non-parental feedings (Prediction 3b) has not been supported by our study. None of the 21 marked magpies that did not successfully rear any chick were observed feeding cuckoo fledglings.

Magpies, in natural non-parasitized populations ignore begging by unrelated fledglings and at the end of the fledgling period become more reluctant to feed their own fledglings in spite of their intensive begging behaviour [76]. Then, why do some magpies in our parasitized population behave “maladaptively” by feeding unrelated parasitic fledglings? In those cases in which magpies are the foster parents that reared the cuckoo chicks in their nests, they continue feeding them after leaving the nest because they learnt the begging calls of those chicks at the end of the nestling period (see references above) and parents are also able to distinguish later the begging calls of their fledglings [77].

Experiments 2 and 3 have demonstrated that some magpies feed foreign cuckoo fledglings, and that alloparental feedings to brood parasitic fledglings are frequently observed (see above). In Experiment 3, when considering only those territories in which we observed non-parental feedings (the adults' point of view) or those in which we observed cuckoo fledglings receiving non-parental feedings (the fledglings' perspective), more than 50% of the feedings consisted of non-parental feedings (see Results, Fig. 2). Although the number of territories in which non-parental feedings were observed was low, probably causing only marginally significant results, these observations do suggest that adults that do provide non-parental feedings do this frequently and, from the fledglings' perspective, that non-parental feedings involve an important source of energy for these fledglings.

Why do some host individuals accept to feed brood parasitic fledglings that were not reared in their nests? The first answer to this question was suggested by Sealy and Lorenzana [38] who proposed that parasitic fledglings may display a supernormal stimulus [78] which would manipulate hosts into feeding them. The best candidate responsible for such a supernormal stimulus would be begging behaviour (see above) because begging is the most important component of avian adult-young communication [79] and begging calls of brood parasitic fledglings are louder, more persistent and more exaggerated than those of host fledglings [20], [37], [70], [71]. However, Experiment 2 has shown that magpie response is highly variable: sometimes they are disposed to feed the stuffed-cuckoo fledgling, but in other cases they show an aggressive behaviour against the stuffed cuckoo, especially dependent on the status of their social group (see above). This result does not support the supernormal stimulus hypothesis because this hypothesis predicts that a supernormal stimulus should be able to manipulate magpie adults into feeding the fledglings regardless the social situation [78].

The second answer to the above-mentioned question is based on the fact that the learning mechanism between parents and offspring at the end of the nestling period usually involves the evolution of highly variable vocal signatures among nestlings that allow parents to recognize and differentiate them later from other fledglings [80]–[82]. We could speculate that great spotted cuckoos developed begging calls that are very attractive to magpies, but characterized by invariable vocal signatures, which would make it difficult for magpies to differentiate between cuckoo fledglings. This scenario would facilitate that any great spotted cuckoo fledgling begging at a high intensity could be fed by magpies that are already feeding other cuckoo fledglings. However, the variability of vocal signatures remains to be studied in fledglings of the great spotted cuckoo and of any other brood parasitic species.

Our results also provide new insights to understand the mechanisms underlying egg or chick discrimination. Magpies are long-lived birds [66]. Therefore, the fact that the current social situation (i.e. caring only for cuckoo fledglings) is a main factor determining the propensity to feed foreign cuckoo fledglings implies that recognition templates (i.e. internal representation of the appearance of parasitic chicks [83]) are not inherited or learned during the female's first breeding attempt as traditional theory assumed [52], [74], [75], [84]–[88] but that they are acquired again at each new breeding attempt, as has been suggested in several more recent studies [89], [90] and has recently experimentally been demonstrated [91].

Magpie nests are frequently multiparasitized (i.e. with more than one great spotted cuckoo egg per nest) either by different females or by the same female laying several eggs in the same nest [58], [92], [93]. The existence of more than one parasitic nestling per nest increases competition and can trigger the starvation of some of them [93]. Now we have two pieces of evidence showing that multiparasitism is selected because of benefits for the cuckoos provided at the fledging stage. Multiparasitism usually prevents survival of any of the host young and this benefits cuckoos during the post-fledgling stage: first, great spotted cuckoo fledglings reared together with magpie nestlings are disadvantaged by magpie foster parents [23], and second, magpies caring for only cuckoo broods are more prone to feed cuckoo fledglings [23], even those that were not reared in their nests (this study).

Interestingly, we have found that male magpies more actively feed their own cuckoo fledglings compared to their female partner. In addition, males, but not females, also tend to feed own cuckoo fledglings more frequently than other unknown cuckoos (Fig. 3). We did however not find any information about sexual differences in feeding frequency of magpie parents to their own fledglings [76], or about feeding frequency by males versus females in any host species of brood parasitic fledglings [20], [22]. It has only been reported that both males and females baywings refused to feed shiny cowbird fledglings [22].

In conclusion, the results obtained in this study are very important for the understanding of the evolution of the arms race between great spotted cuckoos and their magpie hosts because they provide new insights on several relevant points. They indicate that (1) the presence of host's own nestlings for comparison may be a crucial clue favouring the evolution of fledgling discrimination, (2) the benefits provided at the fledging stage could select for multiparasitism, and (3) the results offer new evidence that recognition templates, the basis for the mechanisms underlying egg or chick discrimination, are not inherited or learned during the female's first breeding attempt but are acquired again at each new breeding attempt.

## Supporting Information

Database S1 **Data used for statistical analyses of experiment 1 (translocation of fledglings).**
(PDF)

Database S2 **Data used for statistical analyses of experiment 2 (playback-stuffed-cuckoo presentation).**
(PDF)

Database S3 **Data used for statistical analyses of experiment 3 (non-parental-feeding observations).**
(PDF)
