# Supplementary material for: Great Spotted Cuckoo Fledglings Often Receive Feedings from Other Magpie Adults than Their Foster Parents: Which Magpies Accept to Feed Foreign Cuckoo Fledglings?
Source: PLoS One. 2014 Oct 1;9(10):e107412. doi: 10.1371/journal.pone.0107412 (PMC4182665; doi:10.1371/journal.pone.0107412)
Supplement: Database S1 — Data used for statistical analyses of experiment 1 (translocation of fledglings). (PDF) [file pone.0107412.s001.pdf]

IDChick	Treatment	Survived?
151.759	Control	1
151.699	Control	0
151.780	Control	1
151.440	Control	1
151.170	Control	1
151.720	Control	1
151.318	Control	1
151.800	Traslocated	1
151.040	Traslocated	1
151.340	Traslocated	0
151.380	Control	1
151.400	Traslocated	1
151.360	Traslocated	1
151.230	Traslocated	1
151.230B	Traslocated	1
