# Supplementary material for: Great Spotted Cuckoo Fledglings Often Receive Feedings from Other Magpie Adults than Their Foster Parents: Which Magpies Accept to Feed Foreign Cuckoo Fledglings?
Source: PLoS One. 2014 Oct 1;9(10):e107412. doi: 10.1371/journal.pone.0107412 (PMC4182665; doi:10.1371/journal.pone.0107412)
Supplement: Database S2 — Data used for statistical analyses of experiment 2 (playback-stuffed-cuckoo presentation). (PDF) [file pone.0107412.s002.pdf]

Nest	Year	Treatment	Number of adults present	Approach Behavior	Latency[1]	Number of times adults approached[2]	Number of different adults approaching
CC67	2011	magpie	3	0	2,71	2,35	3
CC3	2011	cuckoo	2	3	1,61	1,87	2
CC27	2011	cuckoo	2	0	NA	0,71	0
CC61	2011	cuckoo	3	0	3,22	1,87	3
CC36	2011	cuckoo	4	0	3,00	2,35	4
CC7	2011	cuckoo	2	0	1,61	3,08	2
CC21	2011	cuckoo	2	0	NA	0,71	0
CC3a	2011	magpie	2	0	NA	1,58	2
CC5a	2011	cuckoo	3	NA	1,79	1,87	3
CC35e	2011	cuckoo	2	3	1,61	1,87	2
CC52	2011	cuckoo	3	2	0,69	1,87	2
ANT18-CC27B	2011	magpie	2	3	1,61	1,22	1
CC14	2011	cuckoo	3	NA	0,69	1,87	3
Ddcha	2011	magpie	1	3	1,39	1,22	1
Dizq	2011	cuckoo	3	2	1,39	1,22	1
D-D	2011	cuckoo	2	2	0,69	1,58	2
A-CC44	2011	cuckoo	2	2	1,10	1,58	2
CC63	2011	cuckoo	2	2	1,10	1,87	2
Pang	2011	magpie	3	NA	0,69	1,87	3
TG-Con	2011	cuckoo	1	2	1,10	1,58	1
FA prof	2011	cuckoo	2	2	2,08	1,58	2
CC18A-CC60A	2011	cuckoo	2	3	1,95	1,87	2
CC63A	2012	cuckoo	1	0	2,48	0,71	1
CC44J	2012	cuckoo	1	2	1,95	1,58	1
CC44I	2012	cuckoo	1	0	3,09	1,22	1
CC51A	2012	cuckoo	1	0	3,14	1,22	1
CC71C	2012	magpie	1	0	2,20	1,22	1
CC19D	2012	magpie	2	3	1,79	1,58	1
CC19H-CC19B	2012	magpie	2	3	2,64	1,58	2
Ddcha2	2012	magpie	1	3	2,48	1,22	1
Dizq2	2012	cuckoo	1	0	2,08	1,22	1

D-D2	2012	cuckoo	2	0	1,95	1,58	2
CC51A-CC52A	2012	cuckoo	2	2	3,09	1,58	2
CC51A-CC52A	2012	magpie	1	3	2,83	1,22	1
FA prof2	2012	cuckoo	3	2	2,64	1,87	3
LaFuen	2012	magpie	2	3	2,48	1,58	2
Tguaj	2012	cuckoo	1	0	2,64	1,22	1
CC66B	2012	cuckoo	2	0	NA	0,71	0
CC33B	2012	cuckoo	3	0	NA	0,71	0
CC37A	2012	cuckoo	2	0	2,30	1,22	2
Abrev	2012	cuckoo	2	0	NA	1,22	1
CC22C	2012	cuckoo	4	3	2,20	2,12	4
Abrev2	2012	cuckoo	8	0	NA	1,87	8
CC72A	2012	cuckoo	2	0	NA	1,22	1
CC31	2012	cuckoo	2	0	NA	1,22	1
CC7B	2012	cuckoo	2	0	NA	1,22	1
CC19i	2012	cuckoo	5	0	2,08	1,22	3
CC2A	2012	cuckoo	4	3	2,77	1,22	1
CC13A	2012	cuckoo	2	3	3,09	1,58	2
CC43AR	2012	cuckoo	4	0	NA	0,71	0
CC40A	2012	cuckoo	2	0	NA	0,71	0
CC38AR	2012	cuckoo	4	0	2,71	1,58	4
CC60	2012	cuckoo	4	3	2,30	3,54	4
CC16C	2012	cuckoo	3	3	3,33	1,22	1
TrasEmi	2012	cuckoo	4	0	NA	0,71	0
CL3B	2012	cuckoo	2	0	NA	0,71	0

1. Log transformed.
2. Sqrt transformed.
