# Supplementary material for: Great Spotted Cuckoo Fledglings Often Receive Feedings from Other Magpie Adults than Their Foster Parents: Which Magpies Accept to Feed Foreign Cuckoo Fledglings?
Source: PLoS One. 2014 Oct 1;9(10):e107412. doi: 10.1371/journal.pone.0107412 (PMC4182665; doi:10.1371/journal.pone.0107412)
Supplement: Database S3 — Data used for statistical analyses of experiment 3 (non-parental-feeding observations). (PDF) [file pone.0107412.s003.pdf]

Territory	Treatment	Ratio nonparental/total feedings provided by adults	Ratio nonparental/total feedings received by fledglings	Parental feedings provided by males per hour	Parental feedings provided by females per hour	Nonparental feedings provided by males per hour	Nonparental feedings provided by females per hour
CC12A	Cuckoo	0,22	0,55	0,78	0,26	0,03	0,26
CC15A	Cuckoo	0,00	0,50	0,51	0,22	0,00	0,00
CC22C	Cuckoo	0,00	0,36	2,12	0,53	0,00	0,00
CC33B	Cuckoo	0,00	0,33	0,53	0,41	0,00	0,00
CC35B	Cuckoo	NA	0,00	NA	NA	NA	NA
CC38HR	Cuckoo	1,00	1,00	0,00	0,00	0,50	1,13
CC39BR	Cuckoo	0,00	0,00	0,43	0,00	0,00	0,00
CC43AR	Cuckoo	0,00	0,77	0,15	0,00	0,00	0,00
CC48AR	Cuckoo	0,00	0,00	0,40	0,35	0,00	0,00
CC60C	Cuckoo	0,60	0,11	1,17	0,00	1,74	0,00
CC66B	Cuckoo	0,61	0,00	0,31	0,23	0,56	0,30
CC7B	Cuckoo	0,50	0,50	0,06	0,00	0,00	0,06
CC16B	Cuckoo	NA	1,00	NA	NA	NA	NA
CC6H	Cuckoo	NA	0,75	NA	NA	NA	NA
CC13A	Mixed	0,00	0,00	NA	NA	NA	NA
CC2A	Mixed	0,00	0,00	NA	NA	NA	NA
CC37A	Mixed	0,00	0,00	NA	NA	NA	NA
CC40A	Mixed	0,00	0,00	NA	NA	NA	NA
